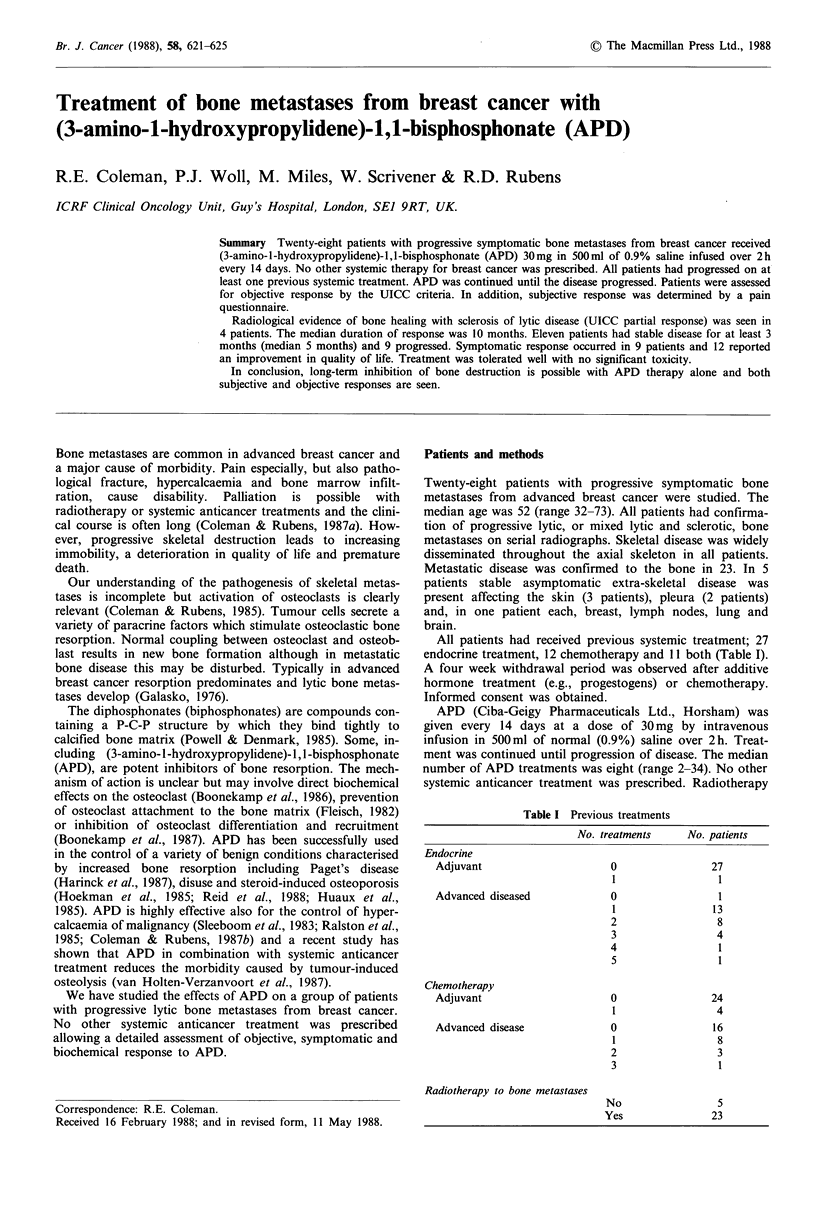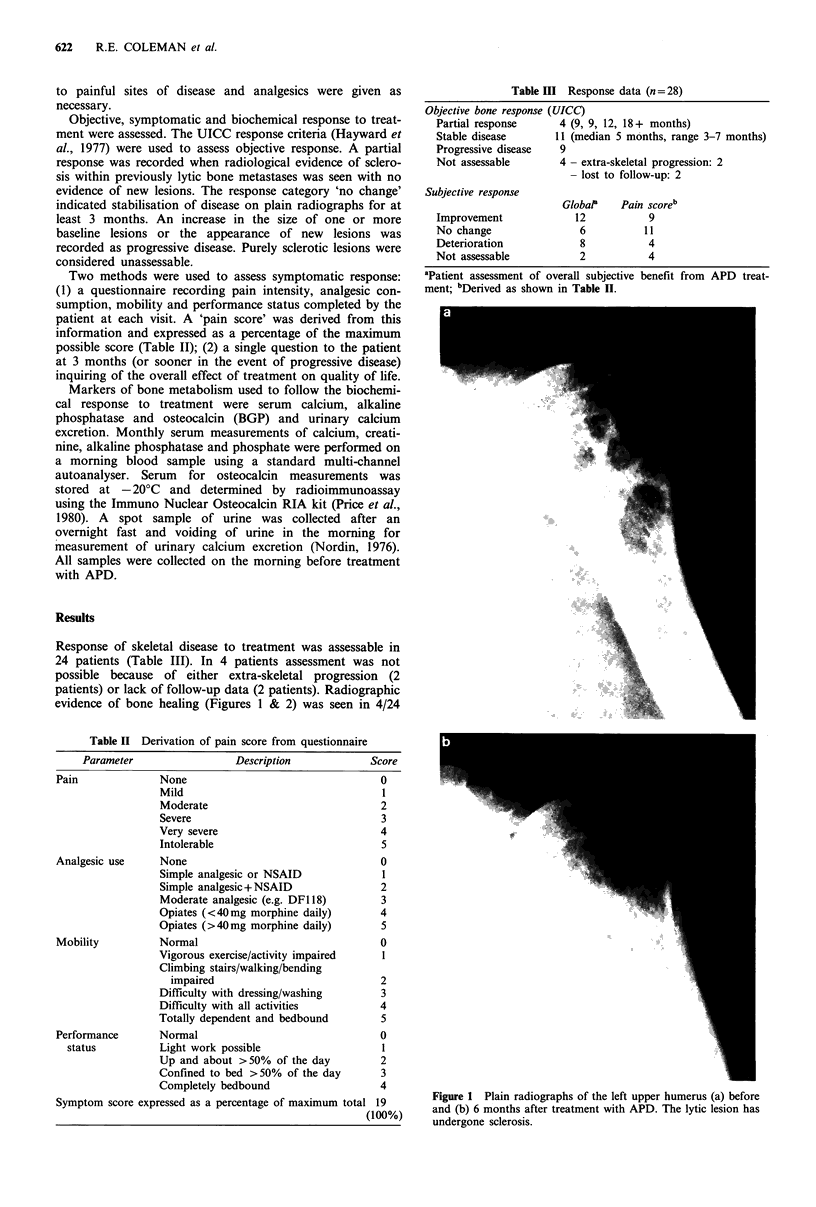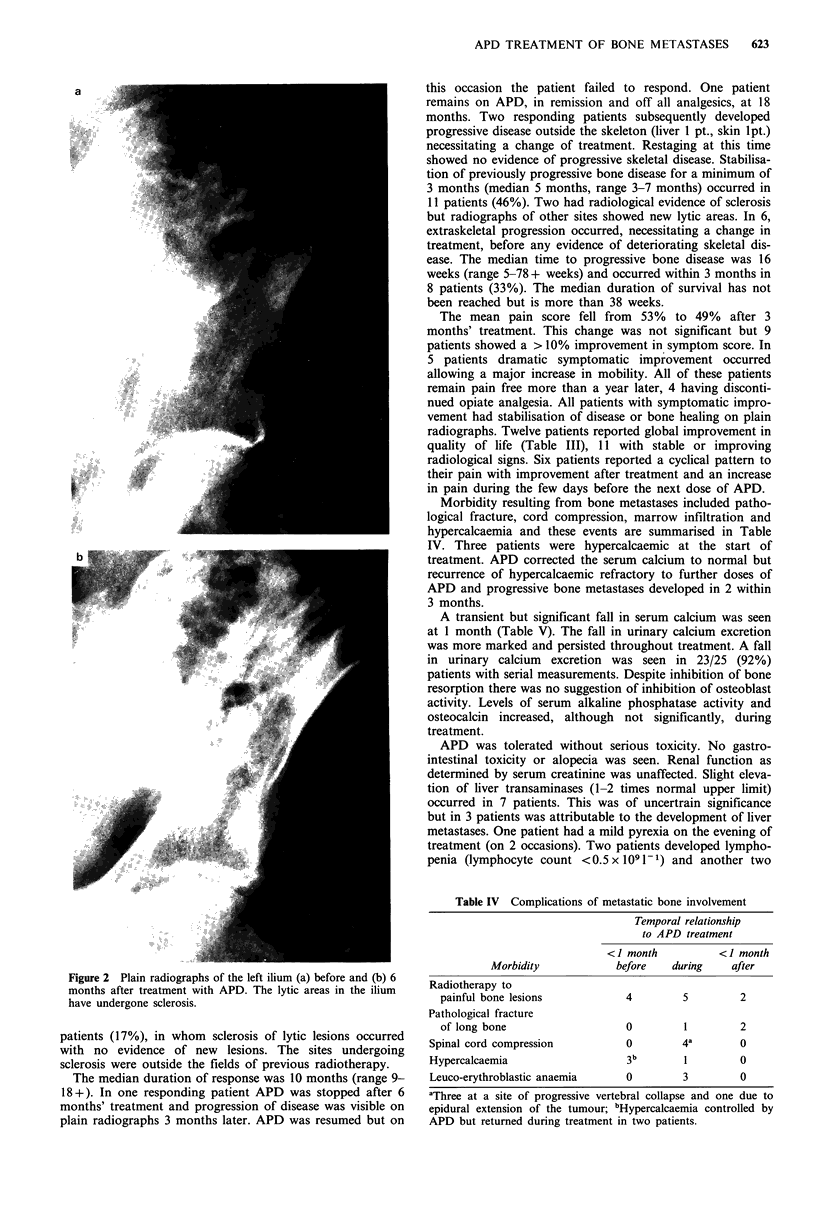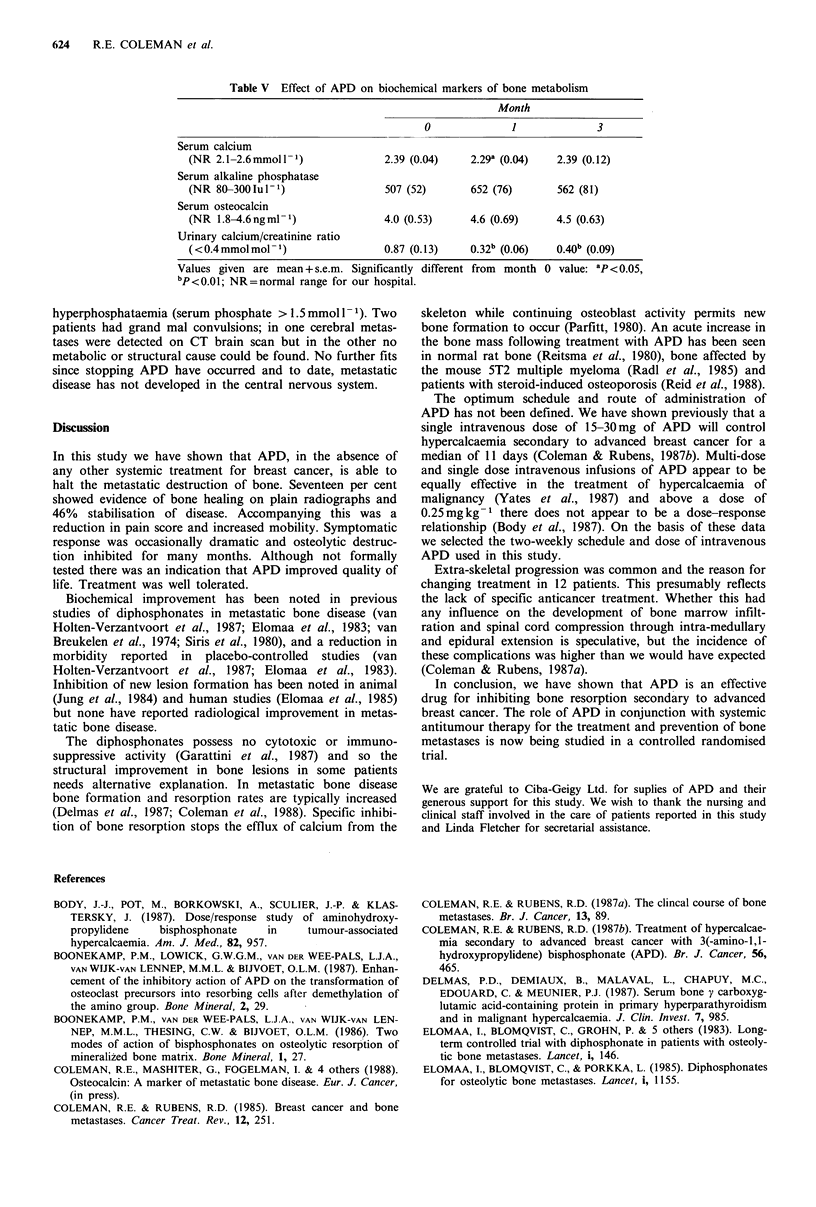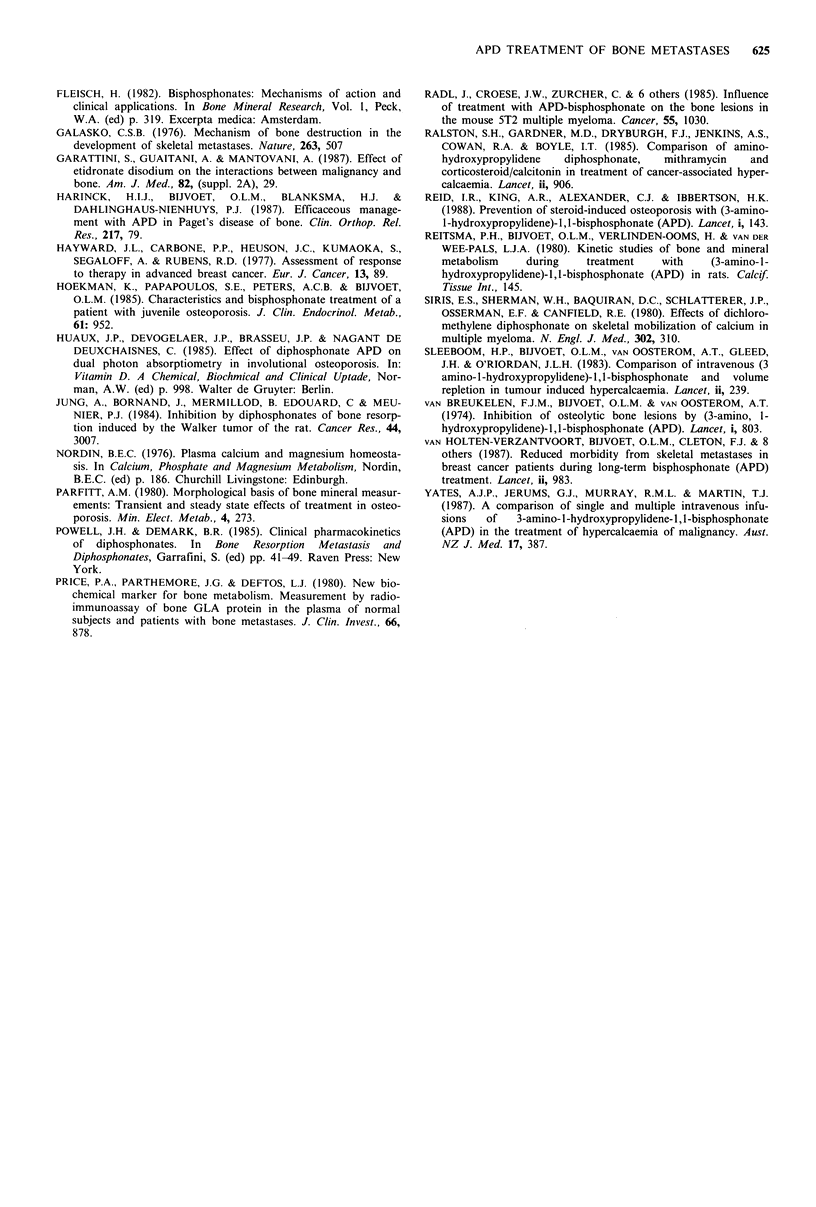# Treatment of bone metastases from breast cancer with (3-amino-1-hydroxypropylidene)-1,1-bisphosphonate (APD).

**DOI:** 10.1038/bjc.1988.272

**Published:** 1988-11

**Authors:** R. E. Coleman, P. J. Woll, M. Miles, W. Scrivener, R. D. Rubens

**Affiliations:** ICRF Clinical Oncology Unit, Guy's Hospital, London, UK.

## Abstract

**Images:**


Br. J. Cancer (1988), 58, 621–625 © The Macmillan Press Ltd., 1988

### Treatment of bone metastases from breast cancer with (3-amino-1-hydroxypropylidene)-1,1-bisphosphonate (APD)

R.E. Coleman, P.J. Woll, M. Miles, W. Scrivener & R.D. Rubens

*ICRF Clinical Oncology Unit, Guy's Hospital, London, SE1 9RT, UK.*

**Summary** Twenty-eight patients with progressive symptomatic bone metastases from breast cancer received (3-amino-1-hydroxypropylidene)-1,1-bisphosphonate (APD) 30 mg in 500 ml of 0.9% saline infused over 2 h every 14 days. No other systemic therapy for breast cancer was prescribed. All patients had progressed on at least one previous systemic treatment. APD was continued until the disease progressed. Patients were assessed for objective response by the UICC criteria. In addition, subjective response was determined by a pain questionnaire.

Radiological evidence of bone healing with sclerosis of lytic disease (UICC partial response) was seen in 4 patients. The median duration of response was 10 months. Eleven patients had stable disease for at least 3 months (median 5 months) and 9 progressed. Symptomatic response occurred in 9 patients and 12 reported an improvement in quality of life. Treatment was tolerated well with no significant toxicity.

In conclusion, long-term inhibition of bone destruction is possible with APD therapy alone and both subjective and objective responses are seen.

Bone metastases are common in advanced breast cancer and a major cause of morbidity. Pain especially, but also pathological fracture, hypercalcaemia and bone marrow infiltration, cause disability. Palliation is possible with radiotherapy or systemic anticancer treatments and the clinical course is often long (Coleman & Rubens, 1987*a*). However, progressive skeletal destruction leads to increasing immobility, a deterioration in quality of life and premature death.

Our understanding of the pathogenesis of skeletal metastases is incomplete but activation of osteoclasts is clearly relevant (Coleman & Rubens, 1985). Tumour cells secrete a variety of paracrine factors which stimulate osteoclastic bone resorption. Normal coupling between osteoclast and osteoblast results in new bone formation although in metastatic bone disease this may be disturbed. Typically in advanced breast cancer resorption predominates and lytic bone metastases develop (Galasko, 1976).

The diphosphonates (biphosphonates) are compounds containing a P-C-P structure by which they bind tightly to calcified bone matrix (Powell & Denmark, 1985). Some, including (3-amino-1-hydroxypropylidene)-1,1-bisphosphonate (APD), are potent inhibitors of bone resorption. The mechanism of action is unclear but may involve direct biochemical effects on the osteoclast (Boonekamp *et al.*, 1986), prevention of osteoclast attachment to the bone matrix (Fleisch, 1982) or inhibition of osteoclast differentiation and recruitment (Boonekamp *et al.*, 1987). APD has been successfully used in the control of a variety of benign conditions characterised by increased bone resorption including Paget's disease (Harinck *et al.*, 1987), disuse and steroid-induced osteoporosis (Hoekman *et al.*, 1985; Reid *et al.*, 1988; Huaux *et al.*, 1985). APD is highly effective also for the control of hypercalcaemia of malignancy (Sleeboom *et al.*, 1983; Ralston *et al.*, 1985; Coleman & Rubens, 1987*b*) and a recent study has shown that APD in combination with systemic anticancer treatment reduces the morbidity caused by tumour-induced osteolysis (van Holten-Verzanvoort *et al.*, 1987).

We have studied the effects of APD on a group of patients with progressive lytic bone metastases from breast cancer. No other systemic anticancer treatment was prescribed allowing a detailed assessment of objective, symptomatic and biochemical response to APD.

Correspondence: R.E. Coleman.
Received 16 February 1988; and in revised form, 11 May 1988.

### Patients and methods

Twenty-eight patients with progressive symptomatic bone metastases from advanced breast cancer were studied. The median age was 52 (range 32–73). All patients had confirmation of progressive lytic, or mixed lytic and sclerotic, bone metastases on serial radiographs. Skeletal disease was widely disseminated throughout the axial skeleton in all patients. Metastatic disease was confirmed to the bone in 23. In 5 patients stable asymptomatic extra-skeletal disease was present affecting the skin (3 patients), pleura (2 patients) and, in one patient each, breast, lymph nodes, lung and brain.

All patients had received previous systemic treatment; 27 endocrine treatment, 12 chemotherapy and 11 both (Table I). A four week withdrawal period was observed after additive hormone treatment (e.g., progestogens) or chemotherapy. Informed consent was obtained.

APD (Ciba-Geigy Pharmaceuticals Ltd., Horsham) was given every 14 days at a dose of 30 mg by intravenous infusion in 500 ml of normal (0.9%) saline over 2 h. Treatment was continued until progression of disease. The median number of APD treatments was eight (range 2–34). No other systemic anticancer treatment was prescribed. Radiotherapy

**Table I** Previous treatments

| | *No. treatments* | *No. patients* |
|---|---|---|
| *Endocrine* | | |
| Adjuvant | 0 | 27 |
| | 1 | 1 |
| Advanced diseased | 0 | 1 |
| | 1 | 13 |
| | 2 | 8 |
| | 3 | 4 |
| | 4 | 1 |
| | 5 | 1 |
| *Chemotherapy* | | |
| Adjuvant | 0 | 24 |
| | 1 | 4 |
| Advanced disease | 0 | 16 |
| | 1 | 8 |
| | 2 | 3 |
| | 3 | 1 |
| *Radiotherapy to bone metastases* | | |
| | No | 5 |
| | Yes | 23 |

to painful sites of disease and analgesics were given as necessary.

Objective, symptomatic and biochemical response to treatment were assessed. The UICC response criteria (Hayward et al., 1977) were used to assess objective response. A partial response was recorded when radiological evidence of sclerosis within previously lytic bone metastases was seen with no evidence of new lesions. The response category 'no change' indicated stabilisation of disease on plain radiographs for at least 3 months. An increase in the size of one or more baseline lesions or the appearance of new lesions was recorded as progressive disease. Purely sclerotic lesions were considered unassessable.

Two methods were used to assess symptomatic response: (1) a questionnaire recording pain intensity, analgesic consumption, mobility and performance status completed by the patient at each visit. A 'pain score' was derived from this information and expressed as a percentage of the maximum possible score (Table II); (2) a single question to the patient at 3 months (or sooner in the event of progressive disease) inquiring of the overall effect of treatment on quality of life.

Markers of bone metabolism used to follow the biochemical response to treatment were serum calcium, alkaline phosphatase and osteocalcin (BGP) and urinary calcium excretion. Monthly serum measurements of calcium, creatinine, alkaline phosphatase and phosphate were performed on a morning blood sample using a standard multi-channel autoanalyser. Serum for osteocalcin measurements was stored at −20°C and determined by radioimmunoassay using the Immuno Nuclear Osteocalcin RIA kit (Price et al., 1980). A spot sample of urine was collected after an overnight fast and voiding of urine in the morning for measurement of urinary calcium excretion (Nordin, 1976). All samples were collected on the morning before treatment with APD.

### Results

Response of skeletal disease to treatment was assessable in 24 patients (Table III). In 4 patients assessment was not possible because of either extra-skeletal progression (2 patients) or lack of follow-up data (2 patients). Radiographic evidence of bone healing (Figures 1 & 2) was seen in 4/24

**Table II** Derivation of pain score from questionnaire

| Parameter | Description | Score |
|---|---|---|
| Pain | None | 0 |
| | Mild | 1 |
| | Moderate | 2 |
| | Severe | 3 |
| | Very severe | 4 |
| | Intolerable | 5 |
| Analgesic use | None | 0 |
| | Simple analgesic or NSAID | 1 |
| | Simple analgesic + NSAID | 2 |
| | Moderate analgesic (e.g. DF118) | 3 |
| | Opiates (<40 mg morphine daily) | 4 |
| | Opiates (>40 mg morphine daily) | 5 |
| Mobility | Normal | 0 |
| | Vigorous exercise/activity impaired | 1 |
| | Climbing stairs/walking/bending impaired | 2 |
| | Difficulty with dressing/washing | 3 |
| | Difficulty with all activities | 4 |
| | Totally dependent and bedbound | 5 |
| Performance status | Normal | 0 |
| | Light work possible | 1 |
| | Up and about >50% of the day | 2 |
| | Confined to bed >50% of the day | 3 |
| | Completely bedbound | 4 |
| Symptom score expressed as a percentage of maximum total | | 19 (100%) |

**Table III** Response data (n = 28)

| | | |
|---|---|---|
| *Objective bone response (UICC)* | | |
| Partial response | 4 (9, 9, 12, 18+ months) | |
| Stable disease | 11 (median 5 months, range 3–7 months) | |
| Progressive disease | 9 | |
| Not assessable | 4 – extra-skeletal progression: 2<br>– lost to follow-up: 2 | |
| *Subjective response* | | |
| | *Global*[a] | *Pain score*[b] |
| Improvement | 12 | 9 |
| No change | 6 | 11 |
| Deterioration | 8 | 4 |
| Not assessable | 2 | 4 |

[a]Patient assessment of overall subjective benefit from APD treatment; [b]Derived as shown in **Table II**.

**Figure 1** Plain radiographs of the left upper humerus (a) before and (b) 6 months after treatment with APD. The lytic lesion has undergone sclerosis.

**Figure 2** Plain radiographs of the left ilium (a) before and (b) 6 months after treatment with APD. The lytic areas in the ilium have undergone sclerosis.

patients (17%), in whom sclerosis of lytic lesions occurred with no evidence of new lesions. The sites undergoing sclerosis were outside the fields of previous radiotherapy.

The median duration of response was 10 months (range 9–18+). In one responding patient APD was stopped after 6 months' treatment and progression of disease was visible on plain radiographs 3 months later. APD was resumed but on this occasion the patient failed to respond. One patient remains on APD, in remission and off all analgesics, at 18 months. Two responding patients subsequently developed progressive disease outside the skeleton (liver 1 pt., skin 1pt.) necessitating a change of treatment. Restaging at this time showed no evidence of progressive skeletal disease. Stabilisation of previously progressive bone disease for a minimum of 3 months (median 5 months, range 3–7 months) occurred in 11 patients (46%). Two had radiological evidence of sclerosis but radiographs of other sites showed new lytic areas. In 6, extraskeletal progression occurred, necessitating a change in treatment, before any evidence of deteriorating skeletal disease. The median time to progressive bone disease was 16 weeks (range 5–78+ weeks) and occurred within 3 months in 8 patients (33%). The median duration of survival has not been reached but is more than 38 weeks.

The mean pain score fell from 53% to 49% after 3 months' treatment. This change was not significant but 9 patients showed a >10% improvement in symptom score. In 5 patients dramatic symptomatic improvement occurred allowing a major increase in mobility. All of these patients remain pain free more than a year later, 4 having discontinued opiate analgesia. All patients with symptomatic improvement had stabilisation of disease or bone healing on plain radiographs. Twelve patients reported global improvement in quality of life (Table III), 11 with stable or improving radiological signs. Six patients reported a cyclical pattern to their pain with improvement after treatment and an increase in pain during the few days before the next dose of APD.

Morbidity resulting from bone metastases included pathological fracture, cord compression, marrow infiltration and hypercalcaemia and these events are summarised in Table IV. Three patients were hypercalcaemic at the start of treatment. APD corrected the serum calcium to normal but recurrence of hypercalcaemic refractory to further doses of APD and progressive bone metastases developed in 2 within 3 months.

A transient but significant fall in serum calcium was seen at 1 month (Table V). The fall in urinary calcium excretion was more marked and persisted throughout treatment. A fall in urinary calcium excretion was seen in 23/25 (92%) patients with serial measurements. Despite inhibition of bone resorption there was no suggestion of inhibition of osteoblast activity. Levels of serum alkaline phosphatase activity and osteocalcin increased, although not significantly, during treatment.

APD was tolerated without serious toxicity. No gastrointestinal toxicity or alopecia was seen. Renal function as determined by serum creatinine was unaffected. Slight elevation of liver transaminases (1–2 times normal upper limit) occurred in 7 patients. This was of uncertrain significance but in 3 patients was attributable to the development of liver metastases. One patient had a mild pyrexia on the evening of treatment (on 2 occasions). Two patients developed lymphopenia (lymphocyte count $<0.5\times10^{9}\,l^{-1}$) and another two

**Table IV** Complications of metastatic bone involvement

| | *Temporal relationship to APD treatment* | | |
|---|---|---|---|
| *Morbidity* | *<1 month before* | *during* | *<1 month after* |
| Radiotherapy to painful bone lesions | 4 | 5 | 2 |
| Pathological fracture of long bone | 0 | 1 | 2 |
| Spinal cord compression | 0 | 4[a] | 0 |
| Hypercalcaemia | 3[b] | 1 | 0 |
| Leuco-erythroblastic anaemia | 0 | 3 | 0 |

[a]Three at a site of progressive vertebral collapse and one due to epidural extension of the tumour; [b]Hypercalcaemia controlled by APD but returned during treatment in two patients.

**Table V** Effect of APD on biochemical markers of bone metabolism

| | *Month* | | |
|---|---|---|---|
| | *0* | *1* | *3* |
| Serum calcium (NR 2.1–2.6 $mmol\,l^{-1}$) | 2.39 (0.04) | 2.29[a] (0.04) | 2.39 (0.12) |
| Serum alkaline phosphatase (NR 80–300 $Iu\,l^{-1}$) | 507 (52) | 652 (76) | 562 (81) |
| Serum osteocalcin (NR 1.8–4.6 $ng\,ml^{-1}$) | 4.0 (0.53) | 4.6 (0.69) | 4.5 (0.63) |
| Urinary calcium/creatinine ratio (<0.4 $mmol\,mol^{-1}$) | 0.87 (0.13) | 0.32[b] (0.06) | 0.40[b] (0.09) |

Values given are mean+s.e.m. Significantly different from month 0 value: [a]$P<0.05$, [b]$P<0.01$; NR = normal range for our hospital.

hyperphosphataemia (serum phosphate $>1.5\,mmol\,l^{-1}$). Two patients had grand mal convulsions; in one cerebral metastases were detected on CT brain scan but in the other no metabolic or structural cause could be found. No further fits since stopping APD have occurred and to date, metastatic disease has not developed in the central nervous system.

### Discussion

In this study we have shown that APD, in the absence of any other systemic treatment for breast cancer, is able to halt the metastatic destruction of bone. Seventeen per cent showed evidence of bone healing on plain radiographs and 46% stabilisation of disease. Accompanying this was a reduction in pain score and increased mobility. Symptomatic response was occasionally dramatic and osteolytic destruction inhibited for many months. Although not formally tested there was an indication that APD improved quality of life. Treatment was well tolerated.

Biochemical improvement has been noted in previous studies of diphosphonates in metastatic bone disease (van Holten-Verzantvoort *et al.*, 1987; Elomaa *et al.*, 1983; van Breukelen *et al.*, 1974; Siris *et al.*, 1980), and a reduction in morbidity reported in placebo-controlled studies (van Holten-Verzantvoort *et al.*, 1987; Elomaa *et al.*, 1983). Inhibition of new lesion formation has been noted in animal (Jung *et al.*, 1984) and human studies (Elomaa *et al.*, 1985) but none have reported radiological improvement in metastatic bone disease.

The diphosphonates possess no cytotoxic or immunosuppressive activity (Garattini *et al.*, 1987) and so the structural improvement in bone lesions in some patients needs alternative explanation. In metastatic bone disease bone formation and resorption rates are typically increased (Delmas *et al.*, 1987; Coleman *et al.*, 1988). Specific inhibition of bone resorption stops the efflux of calcium from the skeleton while continuing osteoblast activity permits new bone formation to occur (Parfitt, 1980). An acute increase in the bone mass following treatment with APD has been seen in normal rat bone (Reitsma *et al.*, 1980), bone affected by the mouse 5T2 multiple myeloma (Radl *et al.*, 1985) and patients with steroid-induced osteoporosis (Reid *et al.*, 1988).

The optimum schedule and route of administration of APD has not been defined. We have shown previously that a single intravenous dose of 15–30 mg of APD will control hypercalcaemia secondary to advanced breast cancer for a median of 11 days (Coleman & Rubens, 1987*b*). Multi-dose and single dose intravenous infusions of APD appear to be equally effective in the treatment of hypercalcaemia of malignancy (Yates *et al.*, 1987) and above a dose of 0.25 $mg\,kg^{-1}$ there does not appear to be a dose–response relationship (Body *et al.*, 1987). On the basis of these data we selected the two-weekly schedule and dose of intravenous APD used in this study.

Extra-skeletal progression was common and the reason for changing treatment in 12 patients. This presumably reflects the lack of specific anticancer treatment. Whether this had any influence on the development of bone marrow infiltration and spinal cord compression through intra-medullary and epidural extension is speculative, but the incidence of these complications was higher than we would have expected (Coleman & Rubens, 1987*a*).

In conclusion, we have shown that APD is an effective drug for inhibiting bone resorption secondary to advanced breast cancer. The role of APD in conjunction with systemic antitumour therapy for the treatment and prevention of bone metastases is now being studied in a controlled randomised trial.

We are grateful to Ciba-Geigy Ltd. for suplies of APD and their generous support for this study. We wish to thank the nursing and clinical staff involved in the care of patients reported in this study and Linda Fletcher for secretarial assistance.

### References

BODY, J.-J., POT, M., BORKOWSKI, A., SCULIER, J.-P. & KLASTERSKY, J. (1987). Dose/response study of aminohydroxypropylidene bisphosphonate in tumour-associated hypercalcaemia. *Am. J. Med.*, **82**, 957.

BOONEKAMP, P.M., LOWICK, G.W.G.M., VAN DER WEE-PALS, L.J.A., VAN WIJK-VAN LENNEP, M.M.L. & BIJVOET, O.L.M. (1987). Enhancement of the inhibitory action of APD on the transformation of osteoclast precursors into resorbing cells after demethylation of the amino group. *Bone Mineral*, **2**, 29.

BOONEKAMP, P.M., VAN DER WEE-PALS, L.J.A., VAN WIJK-VAN LENNEP, M.M.L., THESING, C.W. & BIJVOET, O.L.M. (1986). Two modes of action of bisphosphonates on osteolytic resorption of mineralized bone matrix. *Bone Mineral*, **1**, 27.

COLEMAN, R.E., MASHITER, G., FOGELMAN, I. & 4 others (1988). Osteocalcin: A marker of metastatic bone disease. *Eur. J. Cancer*, (in press).

COLEMAN, R.E. & RUBENS, R.D. (1985). Breast cancer and bone metastases. *Cancer Treat. Rev.*, **12**, 251.

COLEMAN, R.E. & RUBENS, R.D. (1987*a*). The clincal course of bone metastases. *Br. J. Cancer*, **13**, 89.

COLEMAN, R.E. & RUBENS, R.D. (1987*b*). Treatment of hypercalcaemia secondary to advanced breast cancer with 3(-amino-1,1-hydroxypropylidene) bisphosphonate (APD). *Br. J. Cancer*, **56**, 465.

DELMAS, P.D., DEMIAUX, B., MALAVAL, L., CHAPUY, M.C., EDOUARD, C. & MEUNIER, P.J. (1987). Serum bone γ carboxyglutamic acid-containing protein in primary hyperparathyroidism and in malignant hypercalcaemia. *J. Clin. Invest.* **7**, 985.

ELOMAA, I., BLOMQVIST, C., GROHN, P. & 5 others (1983). Long-term controlled trial with diphosphonate in patients with osteolytic bone metastases. *Lancet*, **i**, 146.

ELOMAA, I., BLOMQVIST, C., & PORKKA, L. (1985). Diphosphonates for osteolytic bone metastases. *Lancet*, **i**, 1155.

FLEISCH, H. (1982). Bisphosphonates: Mechanisms of action and clinical applications. In *Bone Mineral Research*, Vol. 1, Peck, W.A. (ed) p. 319. Excerpta medica: Amsterdam.

GALASKO, C.S.B. (1976). Mechanism of bone destruction in the development of skeletal metastases. *Nature*, **263**, 507

GARATTINI, S., GUAITANI, A. & MANTOVANI, A. (1987). Effect of etidronate disodium on the interactions between malignancy and bone. *Am. J. Med.*, **82**, (suppl. 2A), 29.

HARINCK, H.I.J., BIJVOET, O.L.M., BLANKSMA, H.J. & DAHLINGHAUS-NIENHUYS, P.J. (1987). Efficaceous management with APD in Paget's disease of bone. *Clin. Orthop. Rel. Res.*, **217**, 79.

HAYWARD, J.L., CARBONE, P.P., HEUSON, J.C., KUMAOKA, S., SEGALOFF, A. & RUBENS, R.D. (1977). Assessment of response to therapy in advanced breast cancer. *Eur. J. Cancer*, **13**, 89.

HOEKMAN, K., PAPAPOULOS, S.E., PETERS, A.C.B. & BIJVOET, O.L.M. (1985). Characteristics and bisphosphonate treatment of a patient with juvenile osteoporosis. *J. Clin. Endocrinol. Metab.*, **61:** 952.

HUAUX, J.P., DEVOGELAER, J.P., BRASSEU, J.P. & NAGANT DE DEUXCHAISNES, C. (1985). Effect of diphosphonate APD on dual photon absorptiometry in involutional osteoporosis. In: *Vitamin D. A Chemical, Biochmical and Clinical Uptade*, Norman, A.W. (ed) p. 998. Walter de Gruyter: Berlin.

JUNG, A., BORNAND, J., MERMILLOD, B. EDOUARD, C & MEUNIER, P.J. (1984). Inhibition by diphosphonates of bone resorption induced by the Walker tumor of the rat. *Cancer Res.*, **44**, 3007.

NORDIN, B.E.C. (1976). Plasma calcium and magnesium homeostasis. In *Calcium, Phosphate and Magnesium Metabolism*, Nordin, B.E.C. (ed) p. 186. Churchill Livingstone: Edinburgh.

PARFITT, A.M. (1980). Morphological basis of bone mineral measurements: Transient and steady state effects of treatment in osteoporosis. *Min. Elect. Metab.*, **4**, 273.

POWELL, J.H. & DEMARK, B.R. (1985). Clinical pharmacokinetics of diphosphonates. In *Bone Resorption Metastasis and Diphosphonates*, Garrafini, S. (ed) pp. 41–49. Raven Press: New York.

PRICE, P.A., PARTHEMORE, J.G. & DEFTOS, L.J. (1980). New biochemical marker for bone metabolism. Measurement by radioimmunoassay of bone GLA protein in the plasma of normal subjects and patients with bone metastases. *J. Clin. Invest.*, **66**, 878.

RADL, J., CROESE, J.W., ZURCHER, C. & 6 others (1985). Influence of treatment with APD-bisphosphonate on the bone lesions in the mouse 5T2 multiple myeloma. *Cancer*, **55**, 1030.

RALSTON, S.H., GARDNER, M.D., DRYBURGH, F.J., JENKINS, A.S., COWAN, R.A. & BOYLE, I.T. (1985). Comparison of aminohydroxypropylidene diphosphonate, mithramycin and corticosteroid/calcitonin in treatment of cancer-associated hypercalcaemia. *Lancet*, **ii**, 906.

REID, I.R., KING, A.R., ALEXANDER, C.J. & IBBERTSON, H.K. (1988). Prevention of steroid-induced osteoporosis with (3-amino-1-hydroxypropylidene)-1,1-bisphosphonate (APD). *Lancet*, **i**, 143.

REITSMA, P.H., BIJVOET, O.L.M., VERLINDEN-OOMS, H. & VAN DER WEE-PALS, L.J.A. (1980). Kinetic studies of bone and mineral metabolism during treatment with (3-amino-1-hydroxypropylidene)-1,1-bisphosphonate (APD) in rats. *Calcif. Tissue Int.*, 145.

SIRIS, E.S., SHERMAN, W.H., BAQUIRAN, D.C., SCHLATTERER, J.P., OSSERMAN, E.F. & CANFIELD, R.E. (1980). Effects of dichloromethylene diphosphonate on skeletal mobilization of calcium in multiple myeloma. *N. Engl. J. Med.*, **302**, 310.

SLEEBOOM, H.P., BIJVOET, O.L.M., VAN OOSTEROM, A.T., GLEED, J.H. & O'RIORDAN, J.L.H. (1983). Comparison of intravenous (3 amino-1-hydroxypropylidene)-1,1-bisphosphonate and volume repletion in tumour induced hypercalcaemia. *Lancet*, **ii**, 239.

VAN BREUKELEN, F.J.M., BIJVOET, O.L.M. & VAN OOSTEROM, A.T. (1974). Inhibition of osteolytic bone lesions by (3-amino, 1-hydroxypropylidene)-1,1-bisphosphonate (APD). *Lancet*, **i**, 803.

VAN HOLTEN-VERZANTVOORT, BIJVOET, O.L.M., CLETON, F.J. & 8 others (1987). Reduced morbidity from skeletal metastases in breast cancer patients during long-term bisphosphonate (APD) treatment. *Lancet*, **ii**, 983.

YATES, A.J.P., JERUMS, G.J., MURRAY, R.M.L. & MARTIN, T.J. (1987). A comparison of single and multiple intravenous infusions of 3-amino-1-hydroxypropylidene-1,1-bisphosphonate (APD) in the treatment of hypercalcaemia of malignancy. *Aust. NZ J. Med.* **17**, 387.